# Personalized approach of medication by indirect anticoagulants tailored to the patient—Russian context: what are the prospects?

**DOI:** 10.1186/1878-5085-3-10

**Published:** 2012-09-27

**Authors:** Liliya Alexandrovna Belozerceva, Elena Nikolaevna Voronina, Natalia Viktorovna Kokh, Galina Alexandrovna Tsvetovskay, Andrei Pavlovich Momot, Galina Israilevna Lifshits, Maxim Leonidovich Filipenko, Andrei Ivanovich Shevela, Valentin Viktorovich Vlasov

**Affiliations:** 1 Institute of Chemical Biology and Fundamental Medicine, Siberian Branch of the Russian Academy of Sciences (RAS ICBFM), Ac. Lavrentiev 8, Novosibirsk, 630090, Russia

## Abstract

Indirect anticoagulants such as warfarin are the ‘gold standard’ for prevention and treatment of thromboembolic complications in patients at risk (in atrial fibrillation of valvular and nonvalvular etiology, the presence of artificial heart valves, orthopedic and trauma interventions, and other pathological conditions). A wide range of doses required to achieve a therapeutic effect indicates the need for a personalized approach to the appointment of warfarin. In addition to the dependence on the patient's clinical characteristics (sex, age, smoking status, diagnosis), there is a clear association between the warfarin dose and the carriage of certain allelic variants of key genes that makes it possible to apply molecular genetic testing for individual dose adjustment. This provides a more rapid target anticoagulant effect and also reduces the risk of bleeding associated with a possible overdose of warfarin. Implementation of this approach will allow more wide and safe application of indirect anticoagulants in Russia for needy patients.

## Review

## Introduction

**Figure 1:**
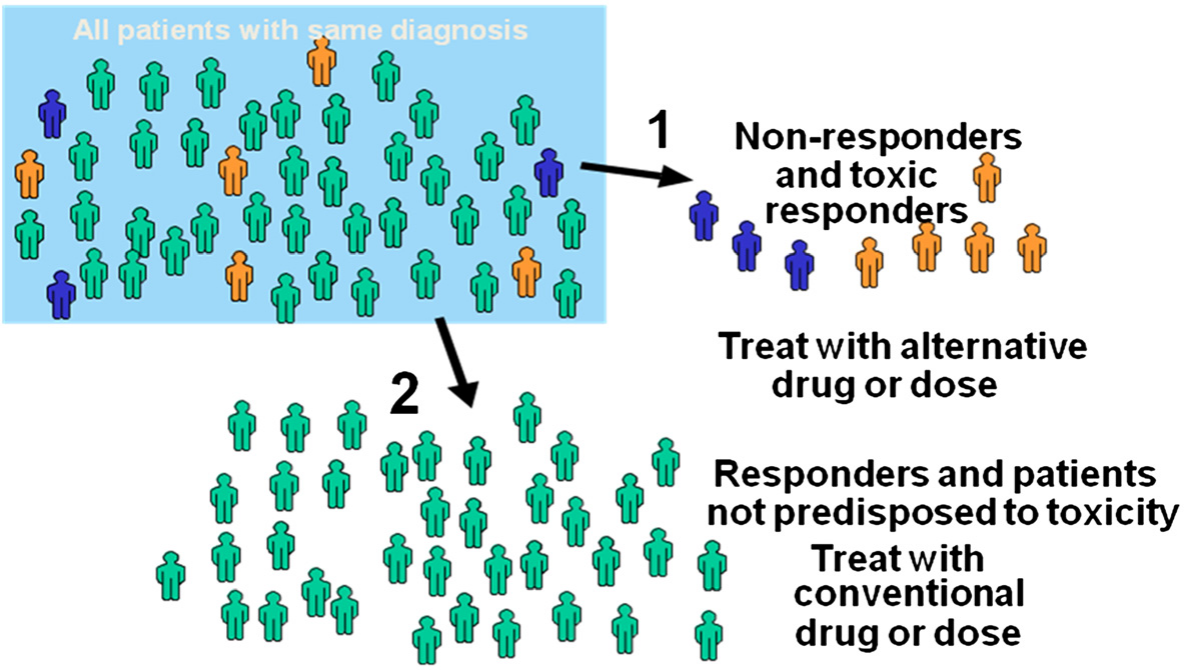
**Clinical potential of pharmacogenomics.** Patients with the same diagnosis are usually treated in the same way, although their responses to drug therapy will not be the same. Pharmacogenetics has the possibility to predict those patients who are likely to have the desired response to the drug, those who are likely to have little or no benefit and those at risk for toxicity (adapted from [1]).

Pharmacogenomics is an important part of personalized medicine which implies that a physician chooses drugs and their doses taking into account individual genetic characteristics of a patient (Figure 1). The danger of receiving indirect anticoagulants is associated with an overdose of the drug that leads to bleeding of varying severity. Moreover, sometimes there is no effect of the treatment in spite of the increase in the dose. Existing methods of the choice of warfarin doses should be improved including molecular genetic analysis of key drug metabolism genes in the diagnostic algorithm.

Warfarin is an oral anticoagulant widely used around the world. For the first time, this drug was synthesized in the laboratory of Karl Link in Wisconsin in 1948, and since the 1950s, it was introduced into clinical practice. Warfarin is commonly used for prevention of thromboembolism in patients with chronic thrombus (blood clot) formed, for example, in atrial fibrillation, prosthetic heart valves and deep venous thrombosis, and for stroke prevention in patients with atrial fibrillation [2,3]. Anticoagulants play a special role in the prevention of thromboembolic complications of cardiovascular diseases. CVD is one of the major targets for predictive, preventive and personalized medicine. A lot of investigations are aimed at identifying risk factors for cardiovascular diseases and prevention strategies [4-7]. For example, in Japan, the combination of public health and personalized treatment activities had contributed to substantial decline in mortality from stroke and ischemic heart disease between the 1960s and 2000s [7].

Thrombosis is a life-threatening condition and one of the main causes of mortality and disability, bringing huge economic loss in Russia. Major complications of anticoagulant therapy are bleeding, which are observed in about 8% of patients receiving warfarin for a year. One percent of them are classified as severe cases (intracranial, retroperitoneal) resulting in hospitalization or transfusion of blood, and 0.25% are fatal cases resulting in the patient's death.

**Figure 2:**
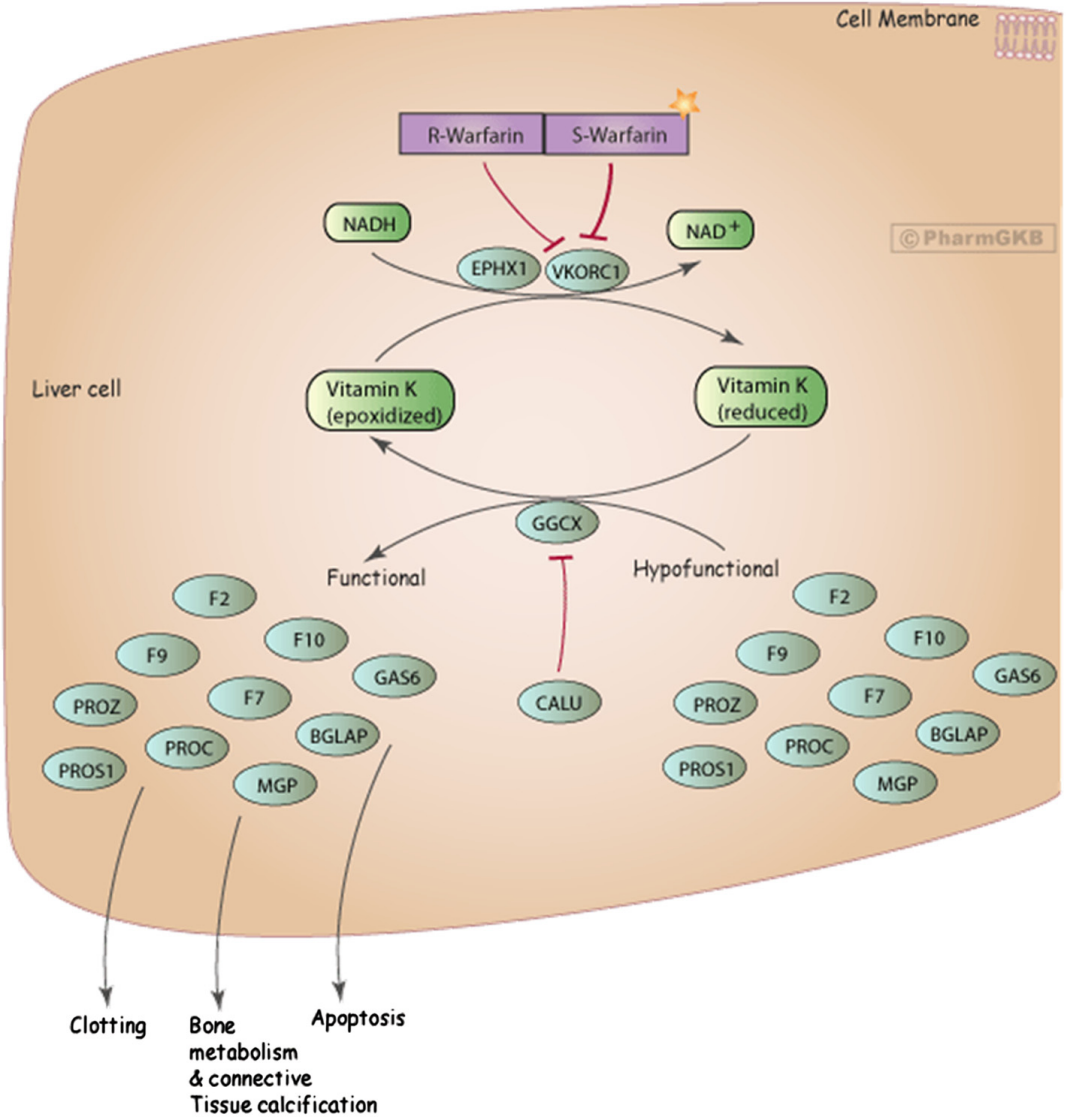
**Warfarin pathway, pharmacodynamics.** The coagulation cascade requires vitamin K in the reduced form as a cofactor for gamma-glutamyl carboxylase to convert inactive factors II, VII, IX, and X to the active forms. Vitamin K is oxidized during this process to vitamin K epoxide. The enzyme VKOR converts vitamin K epoxide back into reduced vitamin K. Warfarin inhibits VKOR, decreasing vitamin K availability, diminishing activatable factors II, VII, IX, and X, and thus inhibiting coagulation (adapted from [2] and [9]).

The molecular target of warfarin is the enzyme vitamin K-epoxide reductase (VKOR), which reduces the oxidized form (epoxide) of vitamin K to hydroquinone (Figure 2). The reduced vitamin K (hydroquinone) is a cofactor of gamma-glutamyl carboxylase (GGCX), which, in turn, provides the carboxylation and, thereby, activates coagulation factors II, VII, IX, and X and proteins C, S, and Z. Warfarin inhibits VKOR that leads to a less reduced form of vitamin K needed for carboxylation of clotting factors [2,8].

**Figure 3:**
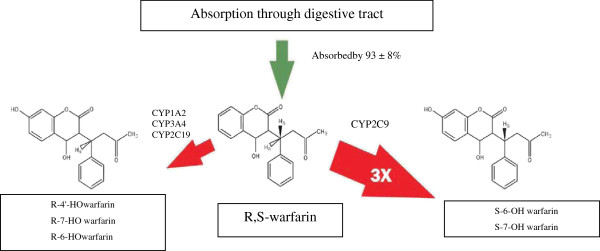
**Metabolism of warfarin.** Warfarin, an enantiomeric mixture of equal concentrations of R- and S-forms, is 93 ± 8% absorbed from the gastrointestinal tract. The rate of metabolism for S-warfarin is approximately three times (3×) faster than that for R-warfarin [2].

Warfarin is a racemic molecule due to the asymmetry of the first aliphatic carbon atom. Clinically available warfarin is a mixture of 50% R- and 50% S-warfarin. Cytochrome P450 2C9 catalyzes the conversion of S-warfarin to an inactive metabolite, 6-hydroxy-S-warfarin, and 7-hydroxy-S-warfarin. CYP 1A1, 2C19, and 3A4 are responsible for metabolism of R-warfarin, which is five times less potent as an inhibitor of VKOR, so its metabolic outcome is much less important in the general scheme of the action of warfarin (Figure 3) [2].

**Figure 4:**
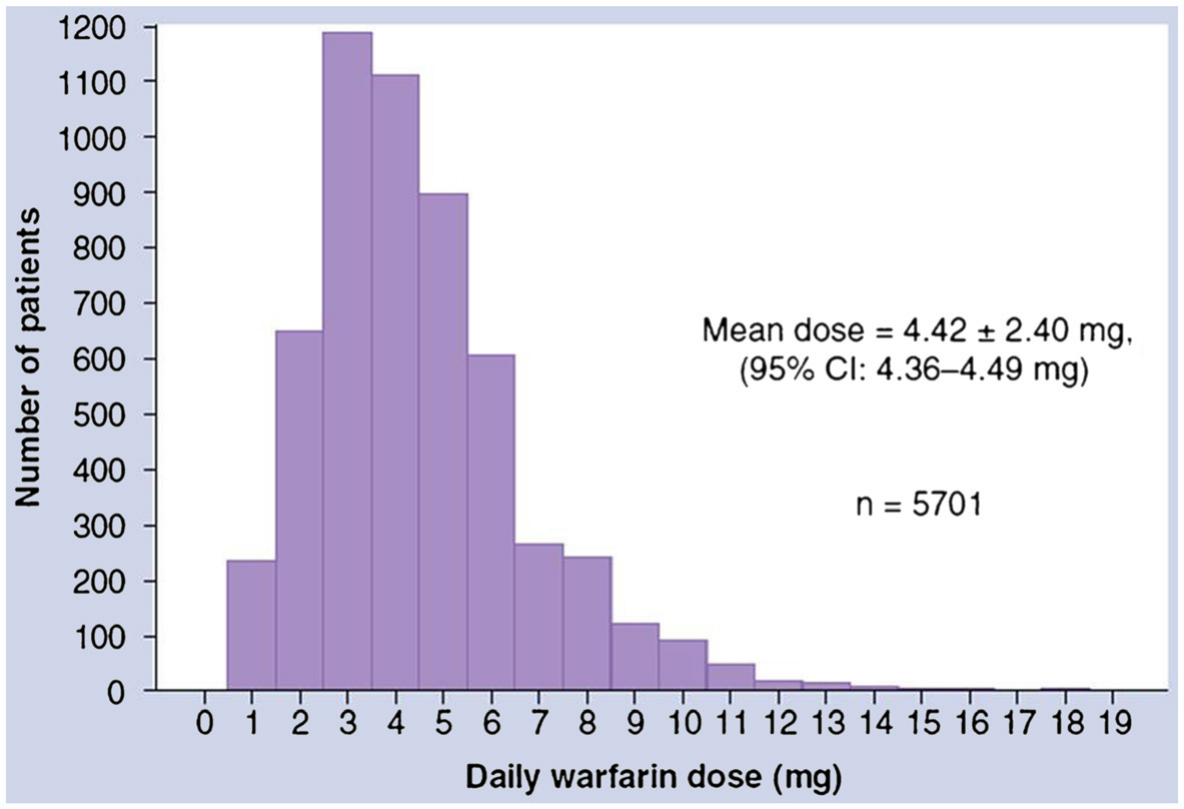
**Variability in warfarin dosingat steady state.** In any given patient population, the daily therapeutic warfarin dose will vary by more than an order of magnitude (adapted from [10]).

Patients show a wide range of interindividual differences in drug doses of warfarin needed to achieve the desired therapeutic effect measured by the international normalized ratio (INR) (Figure 4). This parameter characterizes the patient's plasma clotting time when added to plasma thromboplastin, an activator of blood coagulation. Usually at the beginning of the treatment, a dose of 5 mg/day is assigned; however, the requirement for a dose to achieve the desired INR value may vary from 1 to 20 mg/day. This variation is caused by different factors. The external factors include nutrition, smoking, and concomitant drugs, and the internal ones are genetic factors, age, gender, and body mass index. Environmental and genetic factors cause about 17% and 50% of the variability in warfarin dose, respectively; unknown factors contribute 33% in the variability [2,8].

### Effect of nongenetic parameters

**Figure 5:**
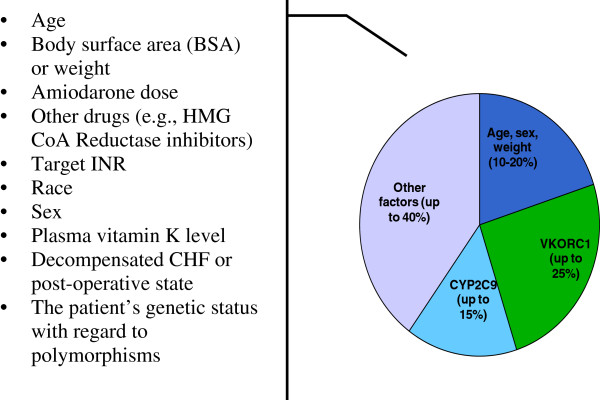
**Factors that correlate withwarfarin dose.** A number of factors affect warfarin dosing, including nongenetic factors (drug-drug interactions, environmental factors, diet, alcohol consumption, and smoking) and genetic factors. Genetic factors explain about 40% of warfarin dosing variability (adapted from [16]).

Easily accessible patient characteristics such as gender, age, body mass index, the interaction with concomitant drugs, smoking, ethnicity, etc., according to some data, provide 20–40% of the variability in warfarin dose (Figure 5). This information is often used for dose selection for patients [11]. It is known that for every 10-kg increase in the patient weight suggests an increase in the daily dose by about 1 mg. According to different studies, the body mass index may explain the warfarin dose variability from 1% (patient group from Sudan, *p* = 0.04) to 2.3% (patient group from China, *p* = 0.026) and 5.7% (European patients) [12-14]. A tendency to reduce the need for warfarin doses is observed with increasing age; this figure may contribute from 2% to 9% in the dose variability [13-15]. Smoking and pulmonary embolism can lead to the increase in the dose of warfarin (*p* = 0.025 and 0.0059, respectively) [15]. The interaction of co-medication is also an important factor affecting the dose that makes 3–4% of interindividual variability [12,14]. According to some studies, amiodarone, which increases the anticoagulant effect of warfarin, may contribute up to 20% in the dose variability (*p* < 0.001) [13].

### Role of polymorphic variants of the *VKORC1* gene

According to literature data, polymorphic variants of the vitamin K-epoxide reductase complex subunit 1 gene (*VKORC1*), which is a target of warfarin, make a maximal contribution to the dose variability of this drug. VKOR is a small (18 kDa, 163 amino acid residues) transmembrane protein of the endoplasmic reticulum; it is expressed mainly in the liver as well as in the heart and pancreas. The *VKORC1* gene encoding vitamin K-epoxide reductase is located at chromosome 16p11.2 and has three exons. Since its opening in 2004, many studies have been performed concerning the influence of *VKORC1* polymorphisms on the need of doses of oral anticoagulants [17,18].

**Figure 6:**
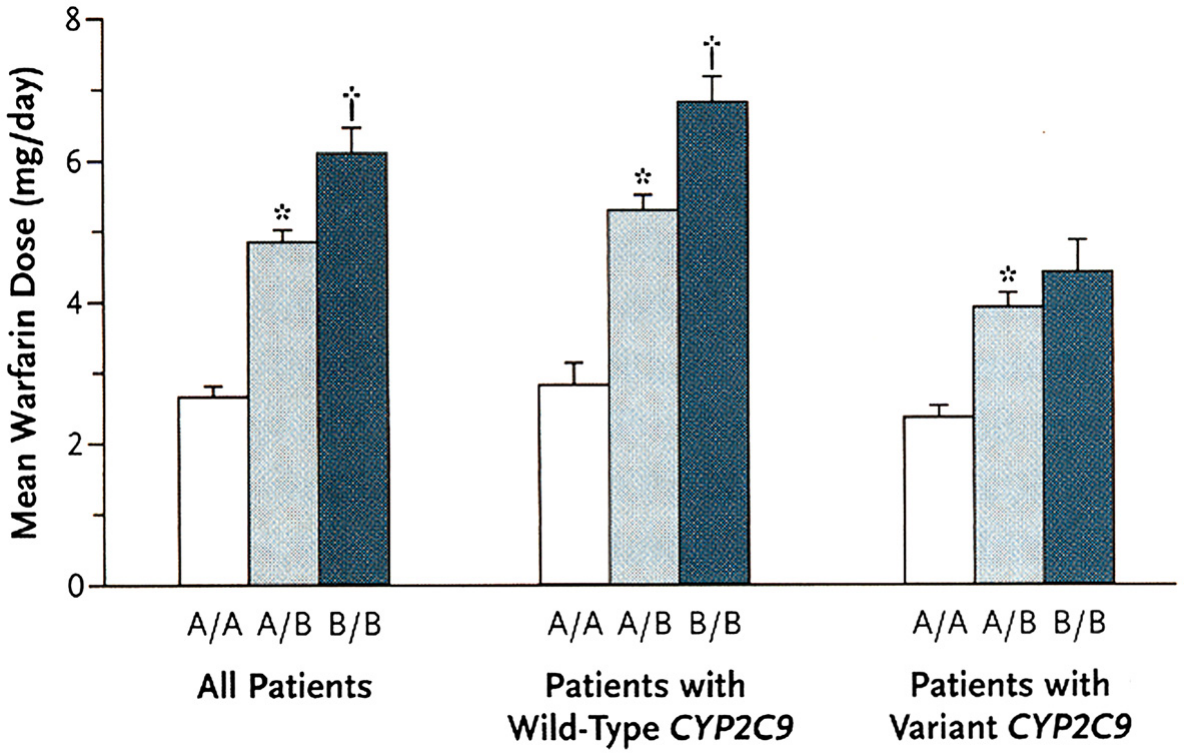
**Effect of VKORC1 haplotypeA or B onwarfarin dosage.** The *asterisks* denote *p* < 0.05 for the comparison with haplotype A/A, and the *daggers* denote *p* < 0.05 for the comparison with haplotype A/B. The *T bars* represent standard errors (adapted from [15]).

Clinically important polymorphic variants of the *VKORC1* gene are -1639G>A (rs9923231) in the promoter region, 1173C>T (rs9934438), 1542G>C (rs8050894), 2255T>C (rs2359612), 3730G>A (rs7294) in introns, and 3^′^-untranslated regions of the gene. These polymorphic loci are in linkage disequilibrium and lead to a change in the amount of produced mRNA [2,14]. Rieder et al. suggest a designation of the -1639A, 1173T, 1542C, 2255C, and 3730A allele combination as haplotype A and the -1639G, 1173C, 1542G, 2255T, and 3730G allele combination as haplotype B [19]. Haplotype A contains allelic variants of the *VKORC1* gene associated with a low expression of the protein that, consequently, requires a smaller concentration of warfarin to achieve a pharmacological effect (Figure 6). The average values of warfarin doses in patients carrying these haplotypes in different races are presented in Table 1.

**Table 1:** **Average dose of warfarinfor patients carrying differenthaplotypes of *VKORC1***

**Number of patients**	**Average dose (mg/day)**		
	***VKORC1*AA**	***VKORC1*AB**	***VKORC1*BB**
603 (White race)	3.1	4.4	5.7
408 (Asian origin)	3.1	3.8	5.6
96 (African origin)	3.7	4.9	6.0

Group A is associated with a low warfarin dose, and group B, with a high warfarin dose [2].

The data show that patients carrying at least one copy of haplotype B receive a higher warfarin dose than homozygous carriers of haplotype A. The dose for the BB genotype is higher by 30% and 80% than that for the AB and AA genotypes, respectively. It is believed that carriers of haplotype B produce two times more mRNA than carriers of haplotype A [2]. D’Andrea et al. studying the influence of polymorphic substitutions in *VKORC1* on the activity of the VKOR protein, did not reveal any alternative mRNA splicing caused by one nucleotide substitution at position 1173 in the *VKORC1* gene. The authors concluded that 1173C>T may be in linkage disequilibrium with other polymorphic variants, which alter the activity of VKOR [20]. Yuan et al. showed that -1639G>A can be that variant [21]. The substitution at position −1639 in the promoter is located in the E-box (CA/GNNTG); another three E-boxes were found a short distance from this place (200 np). The consensus sequence of the E-box is CANNTG. It was shown that E-boxes are important elements for mediating the cell/tissue type-specific transcription, e.g. in the muscles, neurons, liver, and pancreas [22,23]. The replacement of the second base A by G, as it is observed in locus −1639, disturbs the consensus of the E-box and changes the promoter activity. It has been clearly demonstrated that in the HepG2 cell line (human hepatoma cells), the promoter activity was increased by 44% when changing the consensus sequence (A>G). This suggests that the E-box in HepG2 could function as a repressor binding site, and since the line was derived from HepG2 hepatoma, it is likely that the −1639 E-box could also inhibit transcription in the liver [21].

Bodin et al. suggested that the substitution at position −1639 is located in the binding site of transcription factor NF1 (TTGGCCA) [24]. However, the full consensus sequence for NF1 TTGG (A/C) N5GCCAA is not observed near position −1639, and the polymorphic site −1639 is located at the place of the second «N», where any nucleotide is suitable for the binding of the transcription factors. The authors also found no difference in the promoter activity between alleles −1639 A and G [24,25]. However, a number of factors may explain the discrepancy between the results of Bodin et al. and Yuan et al. Cloned promoter regions differ by several base pairs (from −35 to −1798 in the study of Yuan et al. and from −12 to −1756 in the study of Bodin et al.). It should be noted that these authors used different transfection methods: Bodin et al. used calcium phosphate, whereas Yuan et al. used lipofectamine since the calcium phosphate method was less efficient in their study [21,24].

The fact that individuals with the GG genotype of polymorphic locus -1639G>A require a higher dose of warfarin can be explained by the following: the increase in the *VKORC1* promoter activity leads to the enhancement of the mRNA expression from the *VKORC1* gene and, thereby, to increased translation of the VKORC1 protein. The elevated level of mRNA of *VKORC1* can result in the higher VKOR activity and, consequently, enhance the efficiency of the regeneration of reduced vitamin K, which ultimately can provide the higher level of gamma-carboxylation of vitamin K-dependent coagulation factors. The increased number of active clotting factors leads to an increase in the warfarin dose to achieve the anticoagulation effect [17,21].

In the study of Wadelius et al., three polymorphic variants (−1639G>A (rs9923231), 1173C>T (rs9934438), and 2255T>C (rs2359612)) appeared to be the most important for predicting the variability in warfarin dose. The greatest frequency of occurrence of the minor allele in the Caucasoid population is shown to be 0.391 for polymorphic variant -1639G>A. The coefficient of determination (*R*^2^) calculated for a given allele was 0.317, the highest among all other *R*^2^ values calculated for single-nucleotide substitutions in both the *VKORC1* gene and other genes. None of the other ten flanking single-nucleotide substitutions predict an additional variability in dose [14].

The study of patients living in the West Siberian region of Russia showed significant differences in average daily doses of warfarin depending on the VKORC genotype [26]. Among molecular genetic markers, the VKORC genotype makes a maximal contribution in the choice of the therapeutic warfarin dose in the West Siberian population.

### Role of polymorphic variants of the *CYP2C9* gene

Metabolism of warfarin with the CYP2C9 enzyme encoded by the *CYP2C9* gene also influences the warfarin dose. Aithal et al. in 1999, for the first time, reported an association between the *CYP2C9* genotype and the warfarin dose: patients with a low need for warfarin dose (*N* = 36) had one or more allelic variants of *CYP2C9* (compared with patients bearing normal alleles, odd ratio (OR) = 6.2 (95% confidence interval (CI) 2.48–15.6)) [27]. The group of low-dose patients (1.5 mg/day or less) showed more complications during the treatment with warfarin (OR = 5.97, 95% CI 2.26–15.82), and these patients had an increased risk of serious bleeding (rate ratio 3.68, 95% CI 1.43–9.50) compared with a randomly selected control group [27].

**Figure 7:**
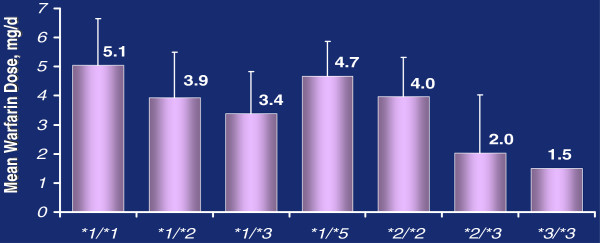
**CYP2C9 polymorphisms and warfarindose.** Bar graph showing the relationship of cytochrome P450 2C9 genotype and warfarin dose. *1/*1 is homozygous wild type and other labels show variants (adapted from [29]).

Several polymorphic variants of the *CYP2C9* gene are identified to reduce the enzyme activity, which leads to an increase in the warfarin concentration in blood serum and frequent bleeding. The CYP2C9*1 allele is considered as the norm [2]. The most common structural polymorphisms of the *CYP2C9* gene are R144C (CYP2C9*2) and I359L (CYP2C9*3). The frequency of the CYP2C9*2 allele is 11–15% in Caucasoid populations and 2–3% in African and Asian populations; the frequency of the CYP2C9*3 allele is 5–7% and 2–4%, respectively [3,28]. According to the majority of data *in vitro*, the presence of allele *2 does not significantly disturb the affinity for the substrate. However, the maximum rate of metabolism (*V*max) reduces by about 50% compared to the level of the CYP2C9*1 allele that leads to a lower rate of warfarin clearance. Amino acid substitution Ile359Leu corresponding to allele *3 results in a significant decrease in the enzyme activity (up to 80%). Carriage of the CYP2C9*2 allele leads to a decrease in warfarin dose by 40% and 68% in heterozygotes and homozygotes, respectively, and carriage of the CYP2C9*3 allele, by 40% and 85%, respectively [2] (Figure 7).

In sum, the cluster of the *CYP2C9* gene on chromosome 10 is the second of the most associated with warfarin dose region after the *VKORC1* gene. This region of loci with high coefficients of linkage disequilibrium includes the *CYP2C9*, *CYP2C8*, *CYP2C18*, and *CYP2C19* genes. Several polymorphic variants of this locus were associated with warfarin dose even after correction for multiple testing [14]. Functional polymorphic variant CYP2C9*3 (rs1057910, Ile359Leu), which greatly limits the hydroxylation of S-warfarin, was the most associated with warfarin dose in this region (*R*^2^ = 0.141) according to Wadelius et al. [14]. Functional polymorphic variant CYP2C9*2 (rs1799853, Arg144Cys), which leads to a moderate decrease in the metabolism of S-warfarin, was not significant in univariate analysis. In this study, the additional genotyping of 53 single-nucleotide substitutions was performed: 17, 10, 14, and 12 in the regions of the *CYP2C9*, *CYP2C19*, *CYP2C18*, and *CYP2C8* genes, respectively. In univariate analysis, a significant association was observed apart from rs1057910 (*3) for nine single-nucleotide polymorphisms (SNPs) including CYP2C19 5^′^ upstream rs3814637 (*R*^2^ = 0.106, minor allele frequency (MAF) = 0.059), the *15 allele and the intronic CYP2C18 rs7896133 (*R*^2^ = 0.074, MAF = 0.056). The CYP2C19*2A allele, which leads to the inactivation of the CYP2C19 enzyme, was not associated with the warfarin dose as in earlier studies [14,30-33].

Alleles *2 and *3 of the*CYP2C9* gene are less common among the African population than among Europeans; CYP2C9*2 is virtually absent in East Asian populations, and *3 allele is also found with a small frequency. However, other polymorphic variants of the *CYP2C9* gene affecting the variability in warfarin dose are found in these populations. In the study of 203 patients from Sudan, the CYP2C9*3 (rs1057910) allele was not found at all, and the CYP2C9*2 allele was revealed at a frequency of approximately 5%. The minor alleles, CYP2C9*5, *8, and *11 were detected at a frequency of 1% each, while CYP2C9*6 and *11, at a frequency of 2% and 5%, respectively. The presence of *2, *5, *6, and *11 alleles decreased the warfarin dose by 16–33% as compared to homozygotes *1/*1, and the dose was not reduced in carriers of *8 and *9 alleles. In this study, polymorphic variants*2, *5, *6, and *11 of the *CYP2C9* gene have made 5% of the variability in warfarin dose in patients (*p* < 0.0001) [12].

In the study of indigenous peoples in Indonesia (*N* = 122), the CYP2C9*2 allele was not identified, and CYP2C9*3 was found with a frequency of 3.6% and had no effect on the warfarin dose (*N* = 85, *p* = 0.9308). In this study, the identified polymorphic variant G/A rs17847036 in the second exon of the *CYP2C9* gene (MAF = 1.8%) contributed to the sensitivity to warfarin with *p* = 0.005. This polymorphic variant along with nongenetic predictors such as age, body mass, and growth of the patients explained 14.5% of interindividual variability in warfarin dose [34].

### Contribution of polymorphic variants of the *CYP4F2* gene

Another gene, *CYP4F2*, which may be considered as a predictor of warfarin dose, encodes cytochrome P450 monooxygenase. Caldwell et al. showed that polymorphic substitution C>T rs2108622 (Val433Met) in the *CYP4F2* gene leads to an increase in the dose requirements of warfarin in the European-American patients. Later, three independent groups of patients showed that this polymorphic variant increases the dose by 1 mg/day in carriers of the T/T genotype compared with C/C (i.e. by 4–12% per C allele) [35].

McDonald et al. proposed a mechanism that explains the effect of the rs2108622 allele on warfarin dose. They showed that the CYP4F2 enzyme functions as vitamin K1-monooxygenase, which generates, probably, omega-hydroxy derivative of the substrate. CYP4F2 may be an important complement to VKOR to limit the excessive accumulation of vitamin K. Carriers of the Val433Met allele of the *CYP4F2* gene have reduced ability to metabolize vitamin K1 due to the rs2108622-dependent reduction of the constant concentration of the enzyme in the liver. Thus, patients with polymorphic variant rs2108622 are predisposed to an increased level of vitamin K1 in the liver and have, therefore, a higher dose requirement for warfarin to achieve therapeutic results. In this study, polymorphic variant Val433Met was significantly associated with the warfarin dose (*p* = 0.02) [36].

The contribution of the *CYP4F2* gene in the variability in warfarin dose was 1–5% according to various sources [13,37]. Not in all cases, however, polymorphic variant rs2108622 considerably influences the dose. In the study of indigenous Indonesians, MAF of the Val433Met allele was 18.85%; however, it was not significantly associated with warfarin dose (*p* = 0.9394) [34].

### Polymorphic variants of other genes

In addition to genes *VKORC1* and *CYP2C9*, polymorphic variants of other genes involved in the warfarin effect may be important, for example, genes *GGCX* (encodes gamma-glutamyl carboxylase), *PROC* (encodes C protein), *EPXH1* (encodes epoxide hydrolase), *FVII* (encodes clotting factor VII), *CALU* (encodes calumenin), and *APOE* (encodes apolipoprotein).

#### Contribution of polymorphic variants of the GGCX gene

Rieder et al. sequenced the *GGCX* gene in 23 Europeans and found 37 single-nucleotide substitutions in this gene, three of which were located at the 5^′^-end of the promoter; five, in the coding region of the gene (two nonsynonymous (Gln325Arg and Leu634Pro) and three synonymous (His194His, Arg406Arg, Thr414Thr)); two, in the 3^′^-nontranslated region; five, at the 3^′^-end of the flanking region; and 22, in introns. Among 21 polymorphic substitutions containing over 5% MAF, six informative substitutions (at positions 4046, 10067, 12970, 13333, 14101, and 14599) were selected for genotyping a sample of Europeans (186 people). The substitution in position 12970 (rs11676382; C/G 11%/89%) in intron 14 of the gene significantly influenced the warfarin dose in all studied models (*p* < 0.05). In this study, polymorphic substitution 12970C>G explained 2% variability in warfarin dose as compared to 21% and 8% for *VKORC1* and *CYP2C9*, respectively. This polymorphism had a small but appreciable effect on warfarin dose (average dose = 5.4 ± 2.6 mg/day (GG) vs. 4.6 ± 2.2 mg/day (CC + CG)), reducing it by 17% [38].

In other studies, polymorphic variant rs12714145 (in position 3261) was also appreciable: it caused 3.3% of the dose variability. It was shown that the dose of warfarin tends to increase with increasing the number of microsatellite repeats in intron 6 GGCX [39-41]. According to Chen et al., haplotype containing microsatellites was more significant than rs12714145 in intron 2, but both of them did not reach statistical significance [14,39-41].

A later study of King et al. in 2010 showed that rs11676382 C>G significantly influenced warfarin dose, while rs12714145 G>A did not. Among 985 White race and African-American patients, 117 (12%) had one or two copies of the G allele of rs11676382. The average values of therapeutic doses of warfarin were 4.9, 4.1, and 3.8 mg/day for C/C, C/G, and G/G, respectively. This single-nucleotide substitution was a significant predictor of dose in this study (*p* = 0.03) and was associated with a decrease in dose by 6.1% per G allele (95% CI 0.6–11.4%). Half of the patients (*n* = 492) had one or two copies of rs12714145 of the A allele. However, this polymorphic substitution was not a significant predictor of dose (*p* = 0.39). The average dose was 4.7 mg/day for A/A or A/G and 5.1 mg/day for G/G [42].

#### Contribution of polymorphic variants of the PROC gene

Protein C (the product of the *PROC* gene) is a vitamin K-dependent serine protease, which destroys factors Va and VIIIa. Antagonists of vitamin K can decrease the activity of protein C and, probably, cause its degradation [43].

Four out of 13 single-nucleotide substitutions in the *PROC* gene analyzed in the study of Wadelius et al. were significantly associated with warfarin dose: rs1799809 A>G (MAF = 0.433) and rs2069901 T>C (MAF = 0.441) in the 5′-regulatory region of *PROC*, rs2069910 C>T (MAF = 0.387) in intron 2, and rs2069919 G>A in intron 3 (MAF = 0.372). Two polymorphic variants in the promoter and one in the third intron achieved significant association with the dose after applying the Bonferroni correction for multiple comparisons within a single gene, which explained 7–9% of differences in warfarin dose (*p* = 0.0002–0.0015). *PROC* haplotypes were also significantly associated with the dose but did not increase statistical significance because the lowest value of *p* was 0.00136 [14].

Carlquist et al. studied the effect of polymorphic variant A/G rs2069919 of the *PROC* gene on warfarin dose in 170 patients and found no significant relationship. Average doses of warfarin for genotypes GG, GA, and AA were 33 ± 16, 32 ± 13, and 29 ± 10 mg/week (medians 30, 28, 26 mg/week), respectively (*p* trend = 0.42; *F* = 0.33, *p* = 0.72) [43]. Polymorphic variants rs2069920, rs1799808, and rs1799809 of the *PROC* gene also showed no effect on warfarin dose in Indonesian patients [34].

#### Contribution of polymorphic variants of the FVII, APOE, and CALU genes

The single-nucleotide substitution in the FVII gene promoter in the position of -402G>A (rs570317) leads to the increase in the transcription rate and the enhanced level of FVII in plasma. Substitution 13407G>A (R353Q, rs6046) in the coding region of the gene leads to the decrease in the expression of the *FVII* gene [44-47]. However, other studies do not confirm the effect of these polymorphic substitutions on warfarin dose [40,48,49]. The authors of works [50-52] describe repeats (four to seven) of 37 np in length in intron 7 of the *FVII* gene. It was shown that the number of these intron repeats directly correlates with the relative expression level of mRNA [50-52]. However, the significant contribution of these repeats in the variability of warfarin dose also was not revealed [48]. Variants of the *FVII* gene resulting in the reduced level of factor FVII may cause the sensitivity to warfarin. Aquilante et al. studied 350 patients of various nationalities and described the influence of polymorphic variant *FVII D/I* (deletion/insertion of 10 bp in the gene promoter in position −323). The average warfarin dose in carriers of D/D was higher by 3.6 mg/week than that in carriers of D/I or I/I; *FVII D/I* contributed 1.3% in the dose variability in this group (*p* = 0.04) [49].

In the study of Wadelius et al., two single-nucleotide substitutions in the *APOE* gene were also analyzed, which determined the commonly used *Е2, *Е, and *Е4 allele systems. Patients who carried the common *E4 allele along with the rarer *E2 allele required higher doses than those who carried *E3 (*p* = 0.0057). This result was significant after the Bonferroni correction for multiple comparisons within the *APOE* gene [14]. It was previously reported that CYP2C9 extensive metabolizers, which were homozygotes APOE*E4, required a higher warfarin dose than other extensive metabolizers (*p* = 0.0008) [53]. In another study, however, APOE*E4 carriers in a homozygous state required a reduced dose of the anticoagulant acenocoumarol [54]. The dependence of warfarin dose on the genotype of polymorphic loci in the *APOE* gene among the Italian population was not also confirmed [55]. These contradictory results show that the association between anticoagulant dose and the *APOE* gene is weak, if any. The allele variants of the genes *GGCX*, *F7*, *PROZ*, *F9*, *EPHX1*, *CALU*, *NR1I2* (encodes the pregnane receptor), and *ORM1* and *ORM2* (encode orosomucoid 1 and 2) had a significant effect on warfarin dose alone but did not pass the correction for multiple testing [14].

The *EPHX1* gene encodes microsomal epoxide hydrolase, the supposed subunit of vitamin K-epoxide reductase, which covers the binding site on the epoxide of vitamin K. The study of Loebstein et al. showed the requirement in high doses of warfarin in CYP2C9-extensive metabolizers carrying nonsynonymous single-nucleotide substitution rs1051740 (Tyr113His) in the *EPHX1* gene. This fact was not, however, confirmed by Wadelius et al., although they showed that the replacement of rs4653436 in *EPHX1* located at the 5^′^-end insignificantly influenced the dose (*p* = 0.00848, *R*^2^ = 0.048) [14,56].

The *CALU* gene encodes the protein calumenin, which binds to vitamin K-epoxide reductase and, probably, inhibits the effect of warfarin [57,58]. Coding single-nucleotide substitution in the *CALU* gene (Arg4Gln), which presumably provides an increase in dose in patients carrying alleles *1/*1 of the *CYP2C9* gene and haplotype BB of the *VKORC1* gene, was not significantly associated with the warfarin dose in the study of Wadelius et al. [14,59]. However, the substitution of rs11653 in the 3^′^-nontranslating region in *CALU*, nonsynonymous rs2307040, and two intron substitutions rs339054 and rs1006023 led to borderline *p* values <0.05. It was shown that there is association of the warfarin dose with rs3762055 located between *ORM1* and *ORM2*, and the haplotype covered this region (*p* = 4.93 × 10^−2^). Finally, a polymorphic Leiden variant in the *FV* gene (rs6025) had no effect on the dose of warfarin in the study of Wadelius et al. (*p* = 0.4925) [14].

### Models of relationship between warfarin dose and genetic and nongenetic parameters

Wadelius et al. conducted an extensive study of variability in genes encoding proteins that are thought to be involved in the action and biotransformation of warfarin. The authors performed genotyping (*N* = 201, European patients) of polymorphic variants in 29 genes whose products are involved in the metabolism of warfarin and examine them for association with warfarin dose. The results showed that polymorphic loci in the genes *VKORC1*, *CYP2C9*, *CYP2C18*, *CYP2C19*, *PROC*, *APOE*, *EPHX1*, *CALU*, *F7*, *GGCX*, *PROZ* (encodes a protein Z), *F9* (encodes coagulation clotting factor IX), *NR1I2*, and *ORM1-ORM2* were significantly related to the dose (*p* < 0.05). Polymorphic variants of the *VKORC1*, *CYP2C9*, *CYP2C18*, and *CYP2C19* genes were significant after applying the Bonferroni correction for multiple testing for all genes studied (*p* < 0.000175). However, association of *CYP2C18* and *CYP2C19* was fully explained by linkage disequilibrium with the loci of CYP2C9*2 and/or *3. *PROC* and *APOE* were both significantly associated with the dose after correction for the number of the studied polymorphic substitutions only within the *APOE* and *PROC* genes, respectively [14].

To explore the possibility of the model of prediction of the warfarin dose, Wadelius et al. combined genes with the greatest influence on warfarin dose (*VKORC1*, *CYP2C9*, and *PROC*) and the characteristics of the patients (age, body mass, interactions with other drugs, and indications for treatment) in a multiple regression model. This model explained 62% of the dose variability. The authors then considered all associated genes *VKORC1*, *CYP2C9*, *CYP2C19*, *CYP2C18*, *PROC*, *APOE*, *EPHX1*, *CALU*, *GGCX*, and *ORM1-2* in the model, which explained 76% of interindividual variability in warfarin dose. Variables with individual *p* values above 0.2 and a low coefficient of determination (*R*^2^) were subsequently removed step-by-step from the model. The achieved model containing *VKORC1*, CYP2C9*2 and *3, *PROC*, *EPHX1*, *GGCX*, *ORM12*, age, body mass, and drug interactions explained 73% of the variability in warfarin dose (Table 2) [14].

**Table 2:** Multiple regression analysis

**Predictor**	**SNP**	**One-dimensional*R***^**2**^	***p***
*VKORC1*	rs9923231	0.317	<0.0001
*CYP2C9*	rs1799853(*2) + rs1057910(*3)	0.159	<0.0001
Age		0.092	0.0029
*PROC*	rs2069919	0.09	0.0416
Body weight		0.057	0.0075
*EPHX1*	rs4653436	0.048	0.1016
Drug interactions		0.036	0.0878
*GGCX*	rs12714145	0.034	0.026
*ORM1*	rs1687390	0.026	0.0571

The analysis explains the 73% variance in warfarin dose in the study of Wadelius et al. [14].

The multiple regression model in experiments in China showed that the *VKORC1* -1639G>A, alleles *CYP2C9* and *EPHX1* 691A>G, as well as age and body mass, explained approximately 76.8% of variability in the dose for Chinese patients having heart valve replacement [60].

On the whole, the combination of VKORC1 -1639G>A, CYP2C9 (*2 and *3), and clinical factors (e.g. age, sex, weight, and amiodarone use) explains approximately 55% of the total variance in warfarin maintenance dose in Caucasians and only about 25% among African-Americans [61-64].

**Figure 8:**
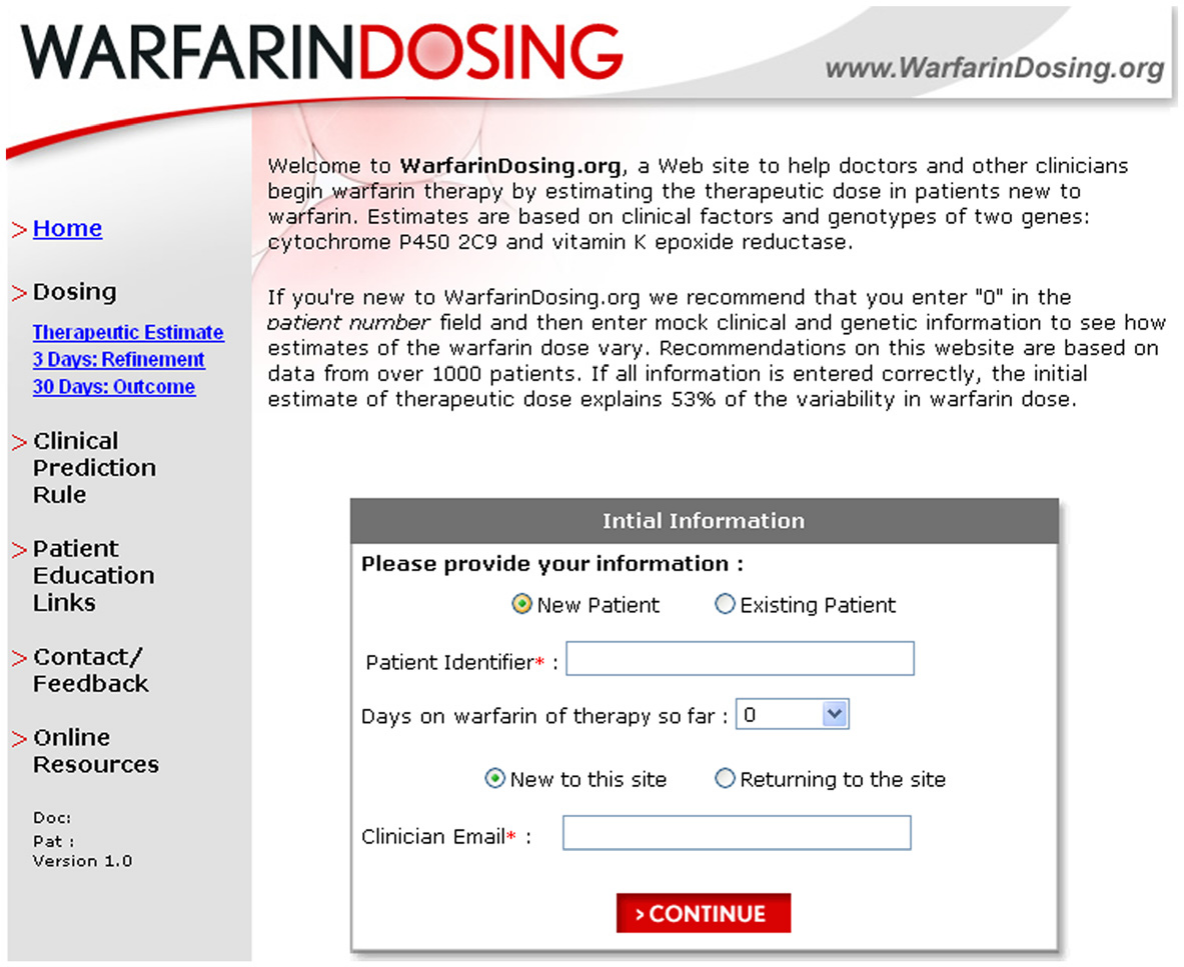
**Online pharmacogenetic algorithm **[65].

The pharmacogenetic algorithm of Gage et al. is widely applicable due to the presence of the online resource [65], which suggests professionals a free use of the technique in exchange for the information about the results (Figure 8) [63]. The equation uses a simple linear relationship; a refinement of the formula is only due to changes in coefficients. However, linear models are rarely observed in living systems. It is necessary to create a new mathematical model, which would completely neutralize the risk of excessive anticoagulation and lead to a significant increase in safety of warfarin therapy.

## Conclusions and recommendations

It is shown that the therapeutic dose of warfarin is influenced by many factors, of which genetic factors contribute more than 50%. Haplotypes A and B of the *VKORC1* gene make the main contribution to the choice of warfarin dose and should be used for the preliminary genetic examination.

In our study, we have registered a significant difference in the average daily dose of warfarin depending on genotype C 1173 T VKORC [26]. The average doses were 7.1 ± 2.3, 4.8 ± 1.9, and 2.8 ± 0.6 mg for patients with the CC (BB), CT (BA), and TT (AA) genotypes, respectively. The presence of ‘slow’ alleles of the *CYP2C9* gene (CYP2C9*2, CYP2C9*3) in a heterozygous state had no reliable effect on the warfarin dose. However, their identification is necessary to assess the risk of bleeding. The influence on the dose is reliable, and the risk of complications significantly increases for carriers of the homozygous ‘slow’ CYP2C9 alleles; however, the frequency of the *2/*2 and *3/*3 genotypes in Western Siberia, to our knowledge, is about 0.5%. If the CYP2C9*2 and CYP2C9*3 alleles are revealed in patients, then patients require more frequent monitoring of INR throughout the warfarin therapy, particularly in the case of the appointment of additional drugs metabolized by cytochrome CYP2C9.

According to our observations, the therapeutic dose of warfarin in carriers of alleles *2,*3 of CYP2C9 is more variable than that in patients with genotype *1*1. The contribution of other genetic markers did not have a significant clinical value in our study. The initial dose of warfarin can be calculated in accordance with the results of pharmacogenetic testing using an online calculator or the ‘Pharmacogenetics’ module of the Russian program PharmSuite [65,66]. The individual initial dose of warfarin is calculated on the basis of the molecular genetic test followed by the adjustment of drug dosage guided by INR and in accordance with the instruction for medical use. The results of the pharmacogenetic testing for CYP2C9 and VKORC1 can predict the fluctuation range of the daily maintaining dose of warfarin and the risk of complications.

According to our data, the pharmacogenetic testing for personalized warfarin dosing can help reduce the time of the dose adjustment, decrease the frequency of episodes of excessive anticoagulation and bleeding by factors of 3 and 4.5, respectively, and minimize the necessity of hospitalization of patients with bleeding and thrombotic complications by 43% and, ultimately, can lower treatment costs. The pharmacogenetic approach for the adjustment of warfarin doses results in the average economic benefit of 1,739.49 rubles (50 €) per patient/year [67]. To confirm the benefits of the pharmacogenetic approach to the dosage of warfarin over the traditional treatment for Russian patients, a large-scale multicenter prospective study is currently being conducted in Russia under the National Project VARFAGEN.

## Practical recommendations

**Figure 9:**
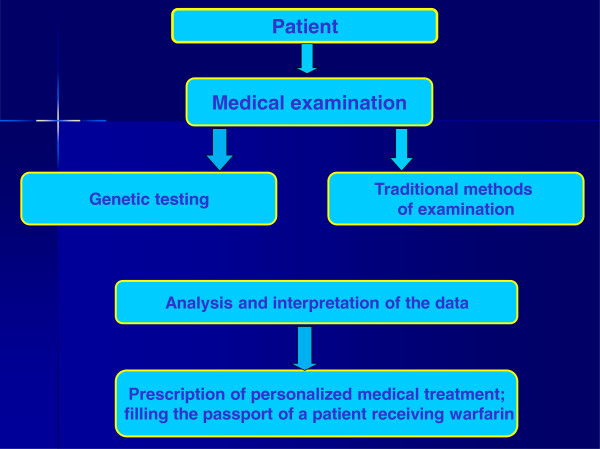
**Algorithm to work withthe challenging group ofpatients.**

It is advisable to recommend the following algorithm for patients, the inhabitants of Western Siberia, which are planned to be treated with warfarin (Figure 9):

1. General clinical testing, evaluation of initial INR, genetic testing of haplotypes A and B of the *VKORC1* gene, and identification of the ‘slow’ alleles CYP2C9*2 and CYP2C9*3 (for 24 h).

2. Calculation of initial and maintenance dose by using an online calculator based on environmental factors (initial and target INR, age, body weight, smoking status, concurrent amiodarone, and/or statins treatment) and identified genetic variants of the *VKORC1* and *CYP2C9* genes.

3. Issuing to the patient a written report containing the calculated dose of warfarin, the frequency of INR monitoring, and recommendations for lifestyle to reduce the risk of side effects. The last section should contain information about the diet features (consumption of vitamin K-containing products and alcohol), the list of drugs, the combination of which could theoretically change the therapeutic dose of warfarin, and recommendation for actions in case of necessary surgical procedures (e.g. tooth extraction).

4. Direct dialling to ‘doctor-patient relationship’.

Coworkers of the Clinical Department in the Center of New Medical Technologies along with the Group of Pharmacogenomics in the Institute of Chemical Biology and Fundamental Medicine of the Siberian Division of RAS have a possibility to adhere to the proposed algorithm that allows one to most efficiently use warfarin and minimize the risk of side effects. To address these issues, we have created the room for patients under warfarin therapy where molecular-genetic and biochemical studies are performed and patients are followed over time.

## Competing interests

The authors declare that they have no competing interests.

## Authors’ contributions

LAB processed the literature and wrote the initial text. ENV selected the scientific articles for review and edited the text. NVK and GAT prepared the clinical recommendations for patients. APM described the relevance of the topic. GIL connected the clinical and scientific articles and edited the general form of the article. MLF edited the general form of the article. AIS helped in the organization of carrying out the research. VVV was the project manager. All authors read and approved the final manuscript.

## Authors’ information

LAB is a student and ENV, Ph.D., is a scientific researcher of the Group of Pharmacogenomics of ICBFM SB RAS. NVK is a scientific researcher in the Laboratory of Personalized Medicine of ICBFM SB RAS. GAT, Ph.D., is a scientific researcher in the Laboratory of Gene Diagnostics of ICBFM SB RAS. APM, M.D., is a professor and the Director of the Hematology Research Center, Altai Branch. GIL, Ph.D., M.D., is the head of the Laboratory of Personalized Medicine of ICBFM SB RAS. MLF, Ph.D., is the head of the Group of Pharmacogenomics of ICBFM SB RAS. AIS is the Deputy Director on Scientific Work of ICBFM SB RAS. VVV is the Director of ICBFM SB RAS.
